# One-piece polarizing interferometer for ultrafast spectroscopic polarimetry

**DOI:** 10.1038/s41598-019-42397-2

**Published:** 2019-04-12

**Authors:** Daesuk Kim, Vamara Dembele

**Affiliations:** Division of Mechanical System Engineering, Chonbuk National University, Jeonju, 54896 Republic of Korea

## Abstract

This paper describes a new class of ultrafast dynamic spectro-polarimetry based on a specially designed one-piece polarizing interferometer. It provides spectral polarimetric parameters of an anisotropic object in milliseconds with high precision. The proposed ultrafast spectro-polarimetry has no moving parts and it is highly robust to external noises. The one-piece polarizing interferometric scheme enables the world fastest and simplest solution in spectroscopic polarimetry. The distinct simple concept on one-piece polarizing interferometer can extract spectroscopic polarimetric parameters Ψ*(k)* and Δ*(k)* precisely with a speed of over 200 Hz over the entire visible wavelength range with a spectral resolution of less than 1 nm. The proposed novel one-piece scheme will have a significant potential of a paradigm shift from lab to fab in polarization metrology.

## Introduction

Polarization characterizing the vectorial nature of electromagnetic wave reveals numerous information on the structure and composition of materials^1–4^, and the nature of scattering and emission^5,6^. Imaging polarimetry has been widely used to measure various information of target object in remote sensing^7^. Polarization also has been applied for engineering light–matter interactions in waveguide^8,9^ and telecommunication^10^. Recently, nanophotonics has opened a new possibility of ultracompact system based on meta-surfaces with a capability of engineering the polarization of light^11–13^. Spectral interferometry employing polarization technique has been playing a very important role in characterizing optical properties in various fields^14–21^. Especially, a variety of spectroscopic polarization measurement solutions have been widely used for material science fields associated with semiconductor and display industry^14–17,22^. Nowadays, spectroscopic ellipsometry (SE) which can provide topographic information relevant to subwavelength periodic structures and thin films has occupied a very important position in semiconductor manufacturing areas^14,22^. Various different types of spectroscopic ellipsometers have been demonstrated^15–18^. Most of them, however, require time-consuming and complex mechanically rotating polarizer or an electrically phase-modulated device to measure the state of polarization of the reflected wave from a measured object. Although the electrically phase-modulated techniques such as photo-elastic modulation, which employs a monochromator based wavelength scanning can provide a millisecond order high-speed ellipsometric parameter measurement capability, it is a speed for a single wavelength measurement and it takes seconds typically to obtain a wide range of ellipsometric parameters Ψ*(k)* and Δ*(k)* for a few hundreds of spectra^16,18^. To overcome such speed limitation, several snapshot spectroscopic polarimetry and ellipsometry have been proposed by replacing the mechanical or electrical modulation mechanism with polarimetric spectral interference based on thick wave-plates^19–21^, birefringent crystal^23^, and multiple spectrometers^24,25^. The channeled spectrum scheme employing multiple thick wave plates has inherent shortcomings such as high spectrum complexity of raw signal resulting in more time-consuming complicated signal processing, reduced spectral resolution, and limited spectral range depending on the availability of thick wave plates. The snapshot spectroscopic polarization measurements employing the dual-spectrum sensing scheme also have an inherent serious drawback of limited vibration robustness resulting in low-precision since it is based on conventional multi-piece interferometry. Moreover, the complicated and bulky schematic with dual spectrometers for snapshot measurement makes it impractical in real applications.

This paper describes a specially designed one-piece polarizing interferometer which enables a new class of ultrafast dynamic spectro-polarimetry. The one-piece polarizing interferometric module needs to be fabricated with special care by mounting all optics in the interferometer tightly as a one-body so that the spectral interference signal with a carrier frequency remains the same all the time even in case the one-piece interferometric module has been shocked by an abrupt external vibration. A key task of the proposed one-piece interferometric scheme is in fixing the spectral carrier frequency created by the optical path difference between the two arms of the one-piece polarizing interferometric module almost perfectly same to extract highly precise spectroscopic polarimetric parameters Ψ*(k)* and Δ*(k)* of an anisotropic object dynamically at over 200 Hz by employing only a single spectrometer. We envision that the uniqueness of the proposed distinct simple one-piece polarizing interferometric concept will stimulate numerous unprecedented novel attempts by which polarization measurement can be moved from lab to fab.

### General principle

#### System design and measurement principle

**Figure 1:**
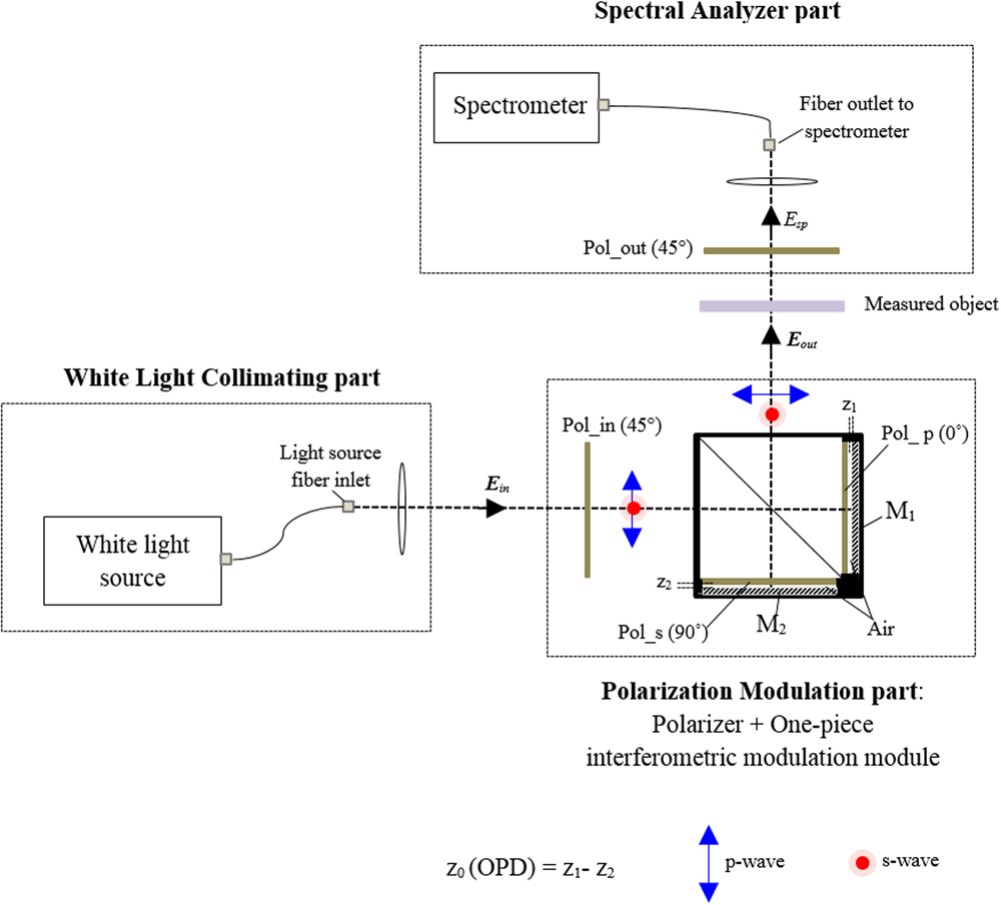
Schematic of the proposed ultrafast spectro-polarimeter based on a one-piece polarizing interferometric module.

The proposed ultrafast spectroscopic polarimeter based on a passive one-piece polarizing Michelson interferometric scheme depicted in Fig. 1 employs only a single spectrometer.

The proposed system is comprised of a collimating part with a white light source and a linear polarizer, a specially designed one-piece polarizing interferometer part, and a single spectrum acquisition part including a linear polarizer. We use a 100 W tungsten-halogen lamp as a light source connected to an optical fiber with a diameter of 1,000 μm. The beam from the white light source passes through the collimating part and the linear polarizer set to 45-degree rotation angle. The linearly polarized beam enters the specially fabricated one-piece polarizing Michelson interferometer module employing a cube type non-polarizing beam splitter, two perpendicularly aligned linear polarizers, and two plane mirrors. The interfered wave modulated by the one-piece polarizing interferometer passes through the measured anisotropic transmissive object, and it enters the single spectrometer having 2047-pixel array with a spectral range of 393 to 647 nm.

We define an input light wave entering the one-piece polarizing interferometer as ***E***_*in*_, and a corresponding output field as ***E***_*out*_, respectively as follows.

1 $${{\boldsymbol{E}}}_{in}(k)=(\begin{array}{c}u(k){e}^{j\xi (k)}\\ v(k){e}^{j\eta (k)}\end{array})$$

2 $${{\boldsymbol{E}}}_{out}(k)={{\boldsymbol{E}}}_{1}(k)+{{\boldsymbol{E}}}_{2}(k)$$

Here, *k* is a wavenumber that equals to 2*π/λ*. *j* refers to a complex operator meaning *j*^2^ = *−*1. *u* and *v* denotes the amplitude of the incident wave. *η* and *ξ* correspond to the phase term of the propagating wave along x- and y-axis, respectively. ***E***_1_*(k)* and ***E***_2_*(k)* correspond to the complex waves travelled through the p- and s- polarization paths of the one-piece polarizing interferometer, respectively. ***E***_1_*(k)* and ***E***_2_*(k)* can be represented as follows.

3 $$\begin{array}{rcl}{{\boldsymbol{E}}}_{1}(k) & = & BP(0){{M}}_{1}P(0)BP(45){{\boldsymbol{E}}}_{in}(k)\\  & = & \frac{1}{\sqrt{2}}[\begin{array}{c}{r}_{1}(u(k){e}^{j[2k{z}_{1}+\xi (k)]}+v(k){e}^{j[2k{z}_{1}+\eta (k)]})\\ 0\end{array}]\\  & = & \frac{1}{\sqrt{2}}[\begin{array}{c}u\text{'}(k){e}^{j[2k{z}_{1}+\xi ^{\prime} (k)]}\\ 0\end{array}]\end{array}$$

And

4 $$\begin{array}{rcl}{{\boldsymbol{E}}}_{2}(k) & = & BP(90){{M}}_{2}P(90)BP(45){{\boldsymbol{E}}}_{in}(k)\\  & = & \frac{1}{\sqrt{2}}[\begin{array}{c}0\\ {r}_{2}(u(k){e}^{j[2k{z}_{2}+\xi (k)]}+v(k){e}^{j[2k{z}_{2}+\eta (k)]})\end{array}]\\  & = & \frac{1}{\sqrt{2}}[\begin{array}{c}0\\ v\text{'}(k){e}^{j[2k{z}_{2}+\eta ^{\prime} (k)]}\end{array}]\end{array}$$

Where, *B* is a Jones matrix of the non-polarizing beam splitter used in the one-piece polarizing interferometer. *P(0)*, *P(45)*, and *P(90)* signify the Jones matrices of the linear polarizers with rotation angles 0°, 45° and 90°, respectively. Note that the two polarizers in the one-piece interferometric module should be aligned to 0 and 90 degrees precisely. *r*_1_ and *r*_2_ denote the complex reflection coefficients of the mirrors *M*_1_ and *M*_2_ used in the interferometer, respectively. *z*_1_ and *z*_2_ indicate optical path length for p- and s- polarization modulation arms, respectively. Here, it is assumed that the optical path difference (OPD) between two paths in the non-polarizing beam splitter is zero. Notably, all components of the one-piece polarizing interferometer are fixed rigidly to maintain the OPD same. *u*′, *ξ*′, and *v*′, *η*′ denote newly defined unknown amplitude and phase terms of ***E***_1_*(k)* and ***E***_2_*(k)*, respectively. Eventually, the inlet wave propagating to the spectrometer *E*_*sp*_*(k)* can be represented as in Eqs (5) and (6): *E*_*sp*_^*ref*^*(k)* means reference complex wave entering the spectrometer through the air where the air is used to get the interfered reference spectrum signal, and *E*_*sp*_^*obj*^*(k)* corresponds to the object complex wave entering the spectrometer after passing through a transmissive anisotropic object as illustrated in Fig. 1, respectively.

5&x02010;1 $${{E}}_{sp}^{ref}(k)={{E}}_{sp}^{1,ref}(k)+{{E}}_{sp}^{2,ref}(k)$$

Where,

5&x02010;2 $$\begin{array}{c}{{E}}_{sp}^{1,ref}(k)=\frac{1}{2}u^{\prime} (k){e}^{j[2k{z}_{1}+\xi ^{\prime} (k)]}\\ {{E}}_{sp}^{2,ref}(k)=\frac{1}{2}v^{\prime} (k){e}^{j[2k{z}_{2}+\eta ^{\prime} (k)]}\end{array}$$

And

6&x02010;1 $${{E}}_{sp}^{obj}(k)={{E}}_{sp}^{1,obj}(k)+{{E}}_{sp}^{2,obj}(k)$$

Where,

6&x02010;2 $$\begin{array}{c}{E}_{sp}^{1,obj}(k)=\frac{1}{2}u^{\prime} (k)|{t}_{p}|{e}^{j[2k{z}_{1}+\xi ^{\prime} (k)+{\delta }_{p}(k)]}\\ {E}_{sp}^{2,obj}(k)=\frac{1}{2}v^{\prime} (k)|{t}_{s}|{e}^{j[2k{z}_{2}+\eta ^{\prime} (k)+{\delta }_{s}(k)]}\end{array}$$

**Figure 2:**
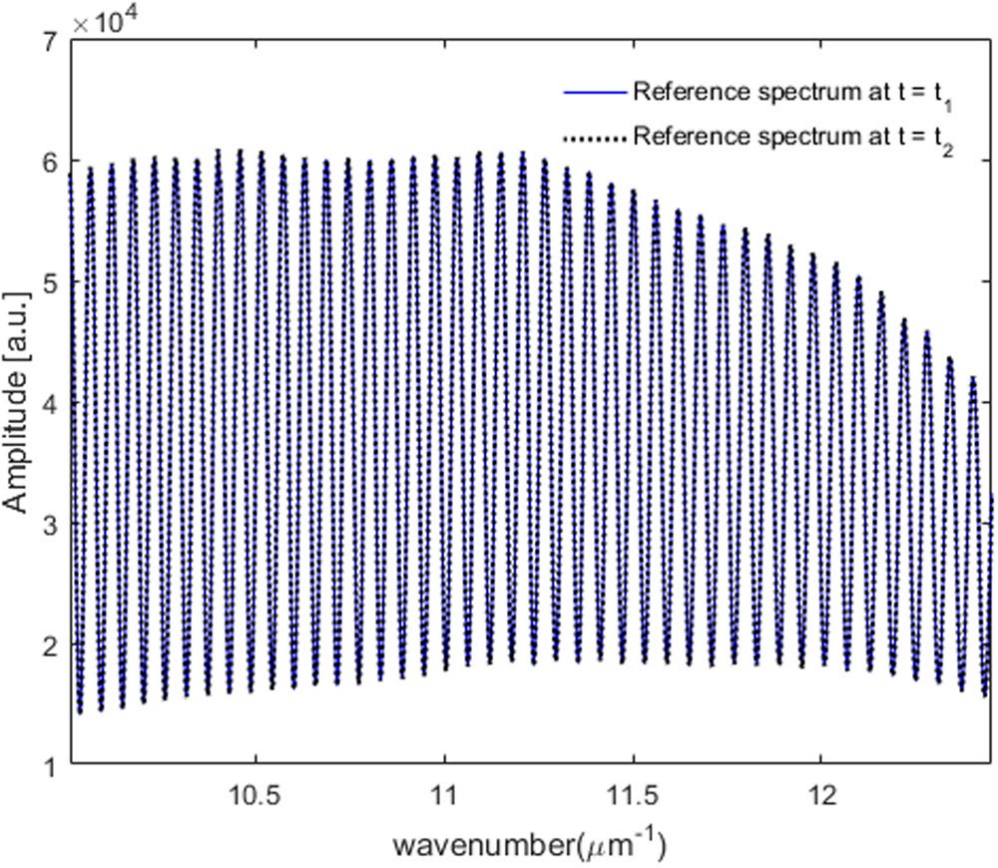
Spectrally interfered reference spectrum recorded at different time measured by the single-channel spectrometer.

Here, *|t*_*p*_*|*, *δ*_*p*_ and *|t*_*s*_*|*, *δ*_*s*_ are the amplitude and the phase change terms of the measured transmissive anisotropic object along p- and s- direction, respectively. The OPD between the two arms of the one-piece polarizing interferometer generates a spectral carrier frequency needed for extracting the spectral polarimetric parameters Ψ*(k)* and Δ*(k)*. Figure 2 shows the raw spectrally interfered reference data acquired by the single-channel spectrometer for the air configuration.

The interfered spectrum measured by a single spectrometer as illustrated in Fig. 2 can be represented as follows.

7 $$I(k)=({{E}}_{sp}^{1}(k)+{{E}}_{sp}^{2}(k)){({{E}}_{sp}^{1}(k)+{{E}}_{sp}^{2}(k))}^{\ast }$$

**Figure 3:**
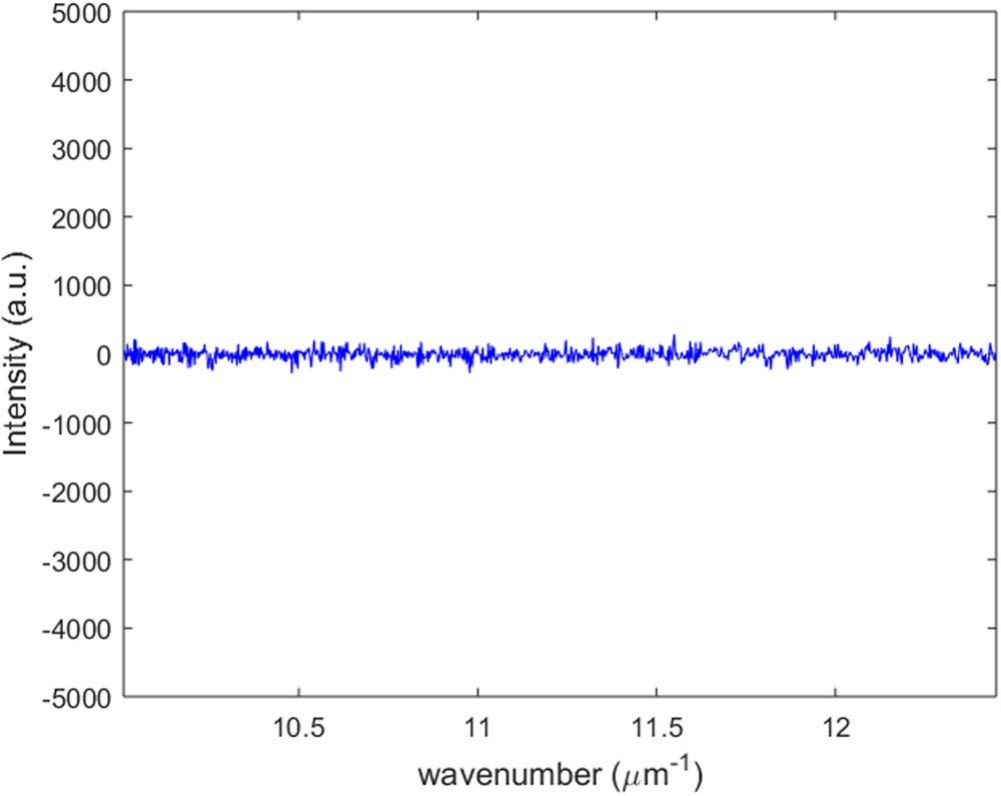
Stability evaluation of the proposed one-piece polarizing interferometer.

The stability of the one-piece polarizing interferometric module is assessed by monitoring the interfered spectrum over sufficiently long time without deliberately applying any disturbance. Note that all experiments have been conducted in general environment without using any optical table. Figure 3 illustrates the noise level obtained by subtracting a spectrum at *t* = *t*_2_ (dark dotted line) from a spectrum at *t* = *t*_1_ (blue solid line) shown in Fig. 2. The mean value of the noise level is around 0.1% of the signal which represents the one-piece interferometric module is fabricated sufficiently firmly to have high robustness to external vibration. As depicted in Fig. 3, the one-piece polarizing interferometer is stable sufficiently to perform the high precision spectral Stokes vector measurement. The noise level fluctuation is associated with light source unstability and electrical noise of the spectrometer rather than mechanical noise in the one-piece polarizing interferometric module.

### Calibration procedure

For dynamic measurement of a spectral Stokes vector of an anisotropic object, we first need a calibration step performed just one time as a pre-preparation procedure. We start the calibration step by measuring a spectrally interfered reference spectrum (raw signal as shown in Fig. 2) for the air configuration which can be represented by Eq. (8).

8 $$\begin{array}{rcl}{I}^{ref}(k) & = & |{E}_{sp}^{1,ref}{|}^{2}+|{E}_{sp}^{2,ref}{|}^{2}+2\gamma |{E}_{sp}^{1,ref}||{E}_{sp}^{2,ref}|\times \,\cos \,{{\rm{\Phi }}}^{ref}(k)\\  & = & {\alpha }^{2}+{\beta }^{2}+2\gamma \alpha \beta \,\cos \,{{\rm{\Phi }}}^{ref}(k)\\ where, &  & {{\rm{\Phi }}}^{ref}(k)=2k{z}_{0}+[\xi \text{'}(k)-\eta \text{'}(k)]\end{array}$$

Here, *γ* represents a spectral coherence function which is systematic. It means that the systematic function is not varied by measured objects. The term *z*_0_ = *z*_1_ − *z*_2_ denotes the optical path difference (OPD) between *z*_1_ and *z*_2_. The OPD *z*_0_ = *z*_1_ − *z*_2_ between the two arms of the one-piece polarizing interferometer should be set to be around 30 to 50 μm for visible range. *Φ*^*ref*^*(k)* denotes the spectral phase function of the interfered reference spectrum *I*^*ref*^*(k)*. *α* and *β* correspond to square root of the DC terms of the complex waves travelling through the p- and s- polarization paths of the one-piece polarizing interferometer, respectively.

**Figure 4:**
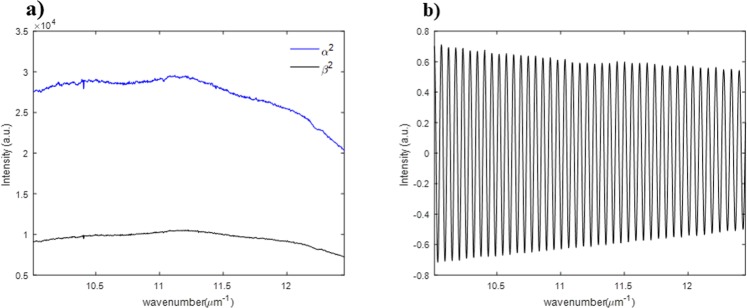
Spectral intensity data required for calibration step: (**a**) measured DC terms and (**b**) modified interfered reference spectrum *I*_*mod*_^*ref*^*(k)*.

**Figure 5:**
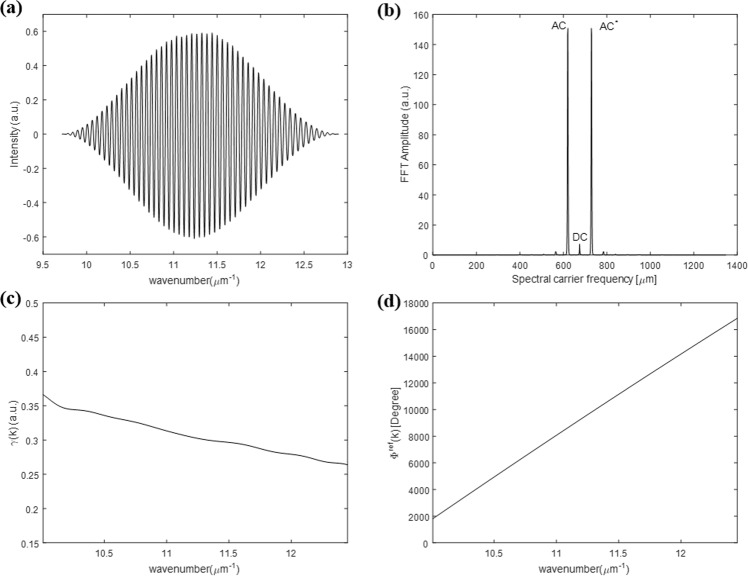
Calibration functions extraction: (**a**) raw spectral data after Hanning function applied to the modified interfered reference spectrum illustrated in Fig. 4(b), (**b**) FFT results of (**a)**, (**c**) extracted spectral coherence function *γ(k)*, and (**d**) extracted spectral phase function *Φ*^*ref*^*(k)* of the reference spectrum.

In order to obtain the amplitude ratio Ψ*(k)* and phase difference Δ*(k)* between p- and s-polarization using a single interfered spectrum of an anisotropic object, we need to extract the spectral coherence function *γ(k)* and the spectral phase function *Φ*^*ref*^*(k)* first. To extract them from the interfered reference spectrum *I*^*ref*^*(k)*, we need to modify the interfered reference spectrum by subtracting *α*^2^ and *β*^2^, and then dividing it by *2αβ* as in Eq. (9). *α* and *β* illustrated in Fig. 4(a) can be measured by using shutters placed at the interferometric arm paths and they are considered to be constant functions when the light source is sufficiently stabilized. Figure 4(b) shows the modified interfered reference spectrum by which we extract the two calibration functions (the spectral coherence function *γ(k)* and the spectral phase function *Φ*^*ref*^*(k)*) simultaneously as illustrated in Fig. 5.

9 $$\begin{array}{rcl}{I}_{{\rm{mod}}}^{ref}(k) & = & \frac{{I}^{ref}(k)-({\alpha }^{2}+{\beta }^{2})}{2\alpha \beta }\\  & = & \gamma (k){\cos {\rm{\Phi }}}^{ref}(k)\end{array}$$

Note again that all this calibration procedure need to be performed just one time prior to the dynamic spectral Stokes vector measurement of anisotropic objects.

After getting the modified interfered reference spectrum depicted in Fig. 4(b), we first multiply the Hanning window to minimize the energy leakage at the edge parts to get the raw signal as illustrated in Fig. 5(a). Then, the Fourier transform method is applied to the Hanning-windowed modified interfered reference spectrum so that we filter out the unwanted DC and one of the AC terms shown in Fig. 5(b). Finally, the spectral coherence function *γ(k)* and the spectral phase function *Φ*^*ref*^*(k)* are extracted as in Fig. 5(c,d) from the amplitude and the phase of the inversely Fourier transformed results of the filtered data in the spectral frequency domain, respectively^26,27^.

## Results: measurement of anisotropic object

### Spectral polarimetric phase Δ(*k*) measurement

**Figure 6:**
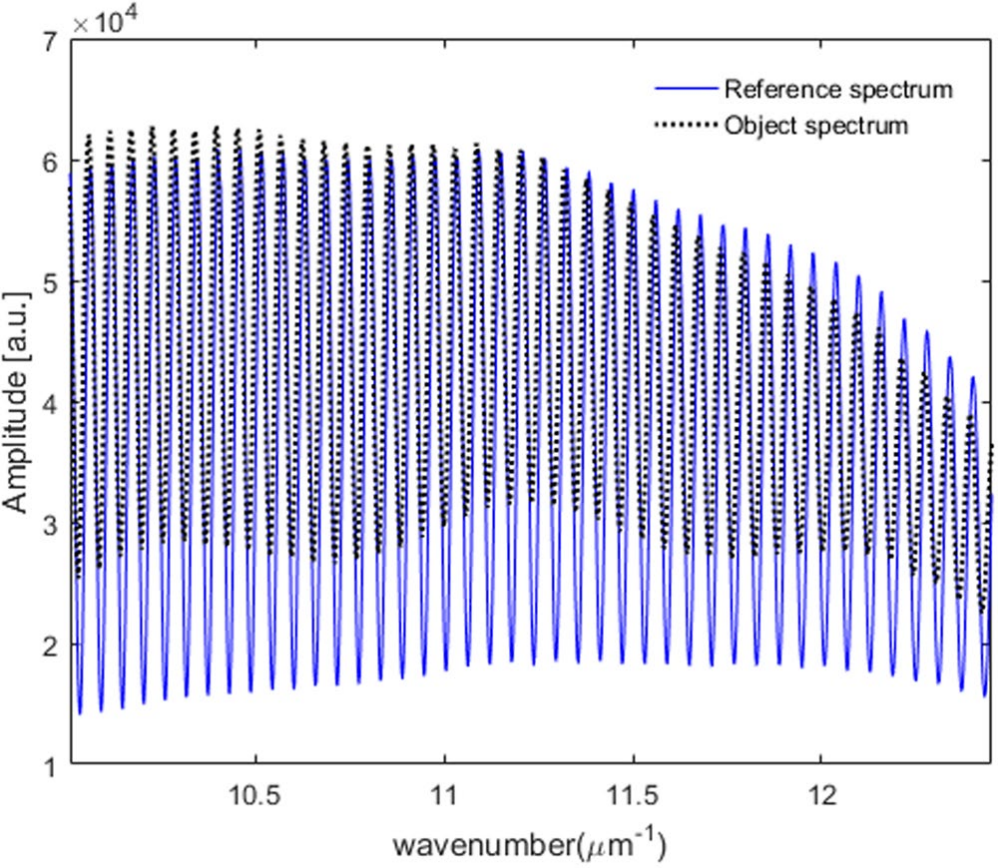
Spectrally interfered object spectrum acquired when a transmissive anisotropic object QWP is placed at the object position (solid blue: reference spectrum, dotted black: object spectrum).

Once we obtain the spectral coherence function *γ(k)* and the spectral phase function *Φ*^*ref*^*(k)* of the interfered reference spectrum through the calibration step, we are ready to measure the amplitude ratio Ψ*(k)* and the phase difference Δ*(k)* between p- and s-polarization by using a single interfered spectrum of an anisotropic object. In this experiment, we use a Quarter Wave Plate (QWP) as a test anisotropic object to show the feasibility of the proposed scheme. When the anisotropic transmissive object is placed at the object position, we acquire the interfered object spectrum as shown in Fig. 6. Figure 6 illustrates the comparison between the spectrally interfered object spectrum with the reference spectrum. We can see that the raw spectrum is changed by the polarimetric characteristics of the measured anisotropic object. The spectrally interfered object spectrum we obtain when an anisotropic object is added to the object position can be represented as in Eq. (10).

10 $$\begin{array}{rcl}{I}^{obj}(k) & = & {(|{E}_{sp}^{1,obj}||{t}_{p}|)}^{2}+{(|{E}_{sp}^{2,obj}||{t}_{s}|)}^{2}+2\gamma |{E}_{sp}^{1,obj}||{E}_{sp}^{2,obj}|\\  &  & \times |{t}_{p}||{t}_{s}|{\cos {\rm{\Phi }}}^{obj}(k)\\  & = & {(\alpha |{t}_{p}|)}^{2}+{(\beta |{t}_{s}|)}^{2}+2\gamma \alpha \beta |{t}_{p}||{t}_{s}|{\cos {\rm{\Phi }}}^{obj}(k)\\ where,{{\rm{\Phi }}}^{obj}(k) & = & 2k{z}_{0}+[\xi \text{'}(k)-\eta \text{'}(k)]+[{\delta }_{p}(k)-{\delta }_{s}(k)]\end{array}$$

The spectral object phase function *Φ*^*obj*^*(k)* can be extracted by using the Fourier transform method in the same way we obtain the spectral reference phase function^26,27^. Eventually, the polarimetric phase difference Δ*(k)* created by the measured anisotropic object is obtained by subtracting *Φ*^*ref*^*(k)* from *Φ*^*obj*^*(k)* since the unknown *2kz*_0_ + *[ξ*′(*k)* − *η*′*(k)]* terms can be removed perfectly.

11 $$\begin{array}{rcl}{\rm{\Delta }}(k) & = & {{\rm{\Phi }}}^{obj}(k)-{{\rm{\Phi }}}^{ref}(k)\\  & = & {\delta }_{p}(k)-{\delta }_{s}(k)\end{array}$$

**Figure 7:**
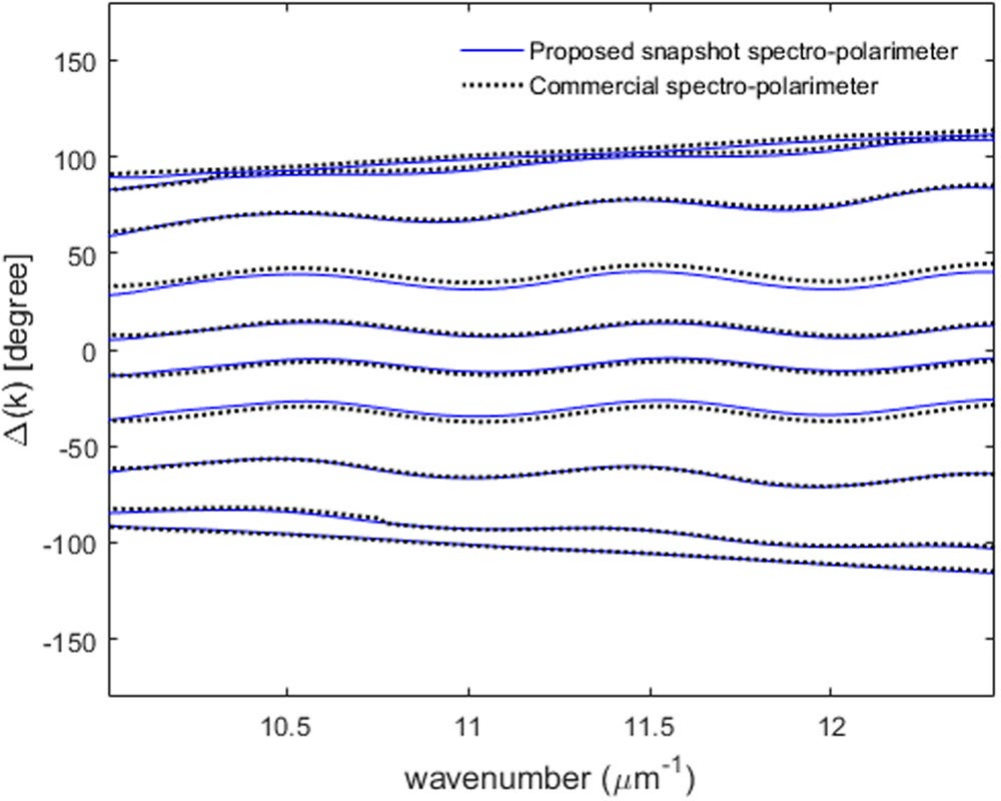
Dynamic Δ*(k)* extraction while the optic axis angle of the QWP is varied from −45° to 45° (solid line: proposed snapshot spectro-polarimeter, and dotted line: commercial spectro-polarimeter).

Figure 7 illustrates Δ*(k)* measurement results for the QWP designed at 632 nm. The solid lines depicted in Fig. 7 show 10 consecutives Δ*(k)* results extracted dynamically by rotating the QWP from −45° to 45° with an interval of 10° by using the proposed one-piece polarizing interferometer based spectroscopic polarimeter. The dotted lines in Fig. 7 illustrate the measurement results by using a commercial spectroscopic polarimeter employing a rotating compensator mechanism which takes around a few seconds to obtain just one Δ*(k)* measurement result. A highly good agreement has been achieved. The comparison results demonstrate that the proposed new concept works correctly and it provides a highly accurate spectral polarimetric phase measurement capability. The sinusoidal oscillation observed in Fig. 7 results from the spectral characteristic of the QWP. To emphasize the measurement speed of the proposed scheme, a video displaying the dynamic Δ*(k)* measurement capability of the QWP object is shown in Supplementary Video S1. The proposed system is extremely robust to external vibration resulting in highly precise Δ*(k)* measurement repeatability of around 0.1° while maintaining dynamic measurement capability. This is a dramatical precision enhancement compared with that of previously proposed complicated snapshot spectro-polarimetry^24,25^.

### Spectral amplitude ratio Ψ*(k)* measurement

To extract Ψ*(k)* simultaneously as well as Δ*(k)* by using the same single object spectrum illustrated in Fig. 6, Eq. (9) is rewritten for simplicity as follows.

12 $${I}^{obj}(k)={A}_{obj}^{DC}+2\gamma {A}_{obj}^{AC}\,\cos \,{{\rm{\Phi }}}^{obj}(k)$$

Here, *A*_*obj*_^*DC*^ = *(α|tp|)*^2^ + (*β|ts|)*^2^ and *A*_*obj*_^*AC*^ = *αβ|tp||ts|*, respectively. *|tp|* and *|ts|* can be extracted by using the two spectral variable functions *A*_*obj*_^*DC*^ and *A*_*obj*_^*AC*^. We can obtain these two spectral variable functions from the object spectrum *I*^*obj*^*(k)* by using the Fourier transform method^26,27^. After obtaining these two spectral variables *A*_*obj*_^*DC*^ and *A*_*obj*_^*AC*^, we can calculate *α|tp|* and *β|ts|* by using the following equations.

13 $$\begin{array}{rcl}\alpha |{t}_{p}| & = & \frac{\sqrt{{A}_{obj}^{DC}+2{A}_{obj}^{AC}}+\sqrt{{A}_{obj}^{DC}-2{A}_{obj}^{AC}}}{2}\\ \beta |{t}_{s}| & = & \frac{\sqrt{{A}_{obj}^{DC}+2{A}_{obj}^{AC}}-\sqrt{{A}_{obj}^{DC}-2{A}_{obj}^{AC}}}{2}\end{array}$$

Since *α* and *β* are two known variables obtained at the calibration step, Ψ*(k)* of the anisotropic object can be extracted directly by using Eq. (14).

14 $$\begin{array}{rcl}{\rm{\Psi }}(k) & = & {\tan }^{-1}(\frac{|{t}_{p}|}{|{t}_{s}|})\\  & = & {\tan }^{-1}(\frac{\beta }{\alpha }\cdot \frac{\sqrt{{A}_{obj}^{DC}+2{A}_{obj}^{AC}}+\sqrt{{A}_{obj}^{DC}-2{A}_{obj}^{AC}}}{\sqrt{{A}_{obj}^{DC}+2{A}_{obj}^{AC}}-\sqrt{{A}_{obj}^{DC}-2{A}_{obj}^{AC}}})\end{array}$$

**Figure 8:**
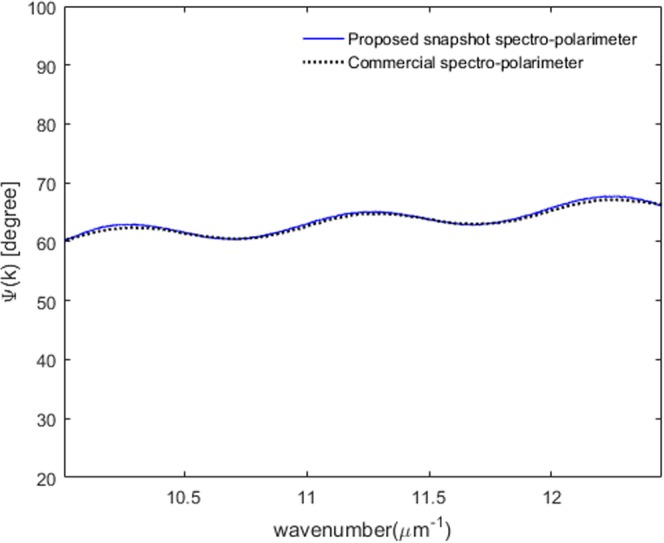
Extraction result of Ψ*(k)* for the QWP with an optic axis angle −15° by using the proposed scheme (solid line: proposed snapshot spectro-polarimeter, dotted line: commercial spectro-polarimeter).

Figure 8 illustrates the extracted Ψ*(k)* for the QWP optic axis angle of −15°. The solid line indicates Ψ*(k)* measured by the proposed snapshot spectro-polarimeter system and the dotted line is that obtained by using a commercial spectroscopic polarimeter. We can see that they are in good agreement for the entire visible range although there are some discrepancies due to limited precision of manual alignment of polarization optics consisting of the system. In summary, we have demonstrated that the spectral polarimetric parameters Ψ*(k)* and Δ*(k)* created by an anisotropic object can be extracted dynamically by using Eqs (11) and (14) once the calibration step is performed prior to the main measurement on an anisotropic object.

### Spectral Stokes vector measurement

Stokes vector represents the polarization state of a propagating wave. Real time Stokes vector measurement for a monochromatic wavelength is well developed and commercially available. However, dynamic spectroscopic polarization measurement has not been demonstrated so far. This paper describes in detail how we can implement such dynamic spectroscopic polarization measurement capability by using a simple one-piece polarizing interferometer module. Note that the proposed scheme cannot be applied for generalized polarization analysis such as scattering in order to have a snapshot measurement capability described in the paper. In Jones matrix representation where we assume perfect polarization, the spectral Stokes vector can be directly calculated by using the following equations^16^.

15 $$(\begin{array}{c}{S}_{1}(k)\\ {S}_{2}(k)\\ {S}_{3}(k)\end{array})=(\begin{array}{c}-\cos \,2{\rm{\Psi }}(k)\\ \sin \,2{\rm{\Psi }}(k)\cos \,{\rm{\Delta }}(k)\\ -\sin \,2{\rm{\Psi }}(k)\sin \,{\rm{\Delta }}(k)\end{array})$$

**Figure 9:**
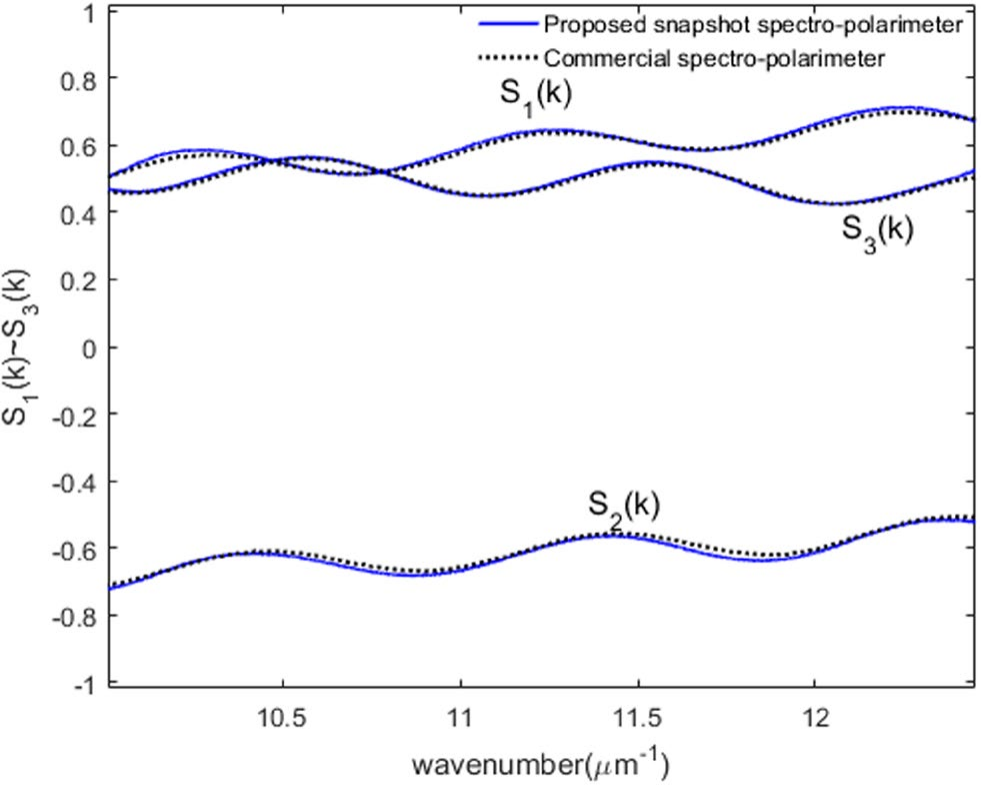
Spectral Stokes vector measurement comparison between the proposed and commercial system when the optic axis angle of a QWP is −15° (solid blue lines: proposed snapshot spectro-polarimeter, black dotted lines: commercial spectro-polarimeter).

As described, since we can extract the spectral amplitude ratio Ψ*(k)* and the spectral phase difference Δ*(k)* between p- and s-polarization just by using a single interfered object spectrum of any anisotropic object, the spectral Stokes vector *S*_1_(*k*), *S*_2_(*k*), and *S*_3_(*k*) can be obtained dynamically by using Eq. (15). Figure 9 illustrates one spectral Stokes vector measurement result obtained by using Ψ*(k)* and Δ*(k)* results for the QWP optics axis of −15°. We can see that the spectral Stokes vector measurement comparison results between the proposed ultrafast snapshot spectro-polarimeter and a commercial rotating analyzer type spectro-polarimeter are in good agreement. Notably, the proposed ultrafast dynamic spectro-polarimetry can measure and display a spectral Stokes vector with around 1,000 spectra at a speed of over 200 Hz with moderate precision and accuracy while the commercial spectro-polarimeter system takes at least seconds typically to obtain the similar level of spectral Stokes vector information.

## Conclusion

A novel ultrafast spectro-polarimeter employing a specially designed highly compact and robust one-piece polarizing interferometric module has been described. In this paper, we have successfully extracted the spectral polarimetric parameters Ψ*(k)* and Δ*(k)* in milliseconds from which accurate spectral Stokes vector with over 1,000 spectra can be measured dynamically. We envision that the proposed distinct simple but powerful one-piece polarizing interferometer can be extended to numerous applications where ultrafast spectroscopic polarization measurement capability becomes critical and inevitable such large-scale R2R thin film coating and nano-patterning in-line monitoring for flexible electronics and optical film devices.

## Supplementary information

Supplementary Video for ′One-piece polarizing interferometer for ultrafast spectroscopic polarimetry′

Supplementary information for the Supplementary Video
