# Supplementary material for: One-piece polarizing interferometer for ultrafast spectroscopic polarimetry
Source: Sci Rep. 2019 Apr 12;9:5978. doi: 10.1038/s41598-019-42397-2 (PMC6461686; doi:10.1038/s41598-019-42397-2)
Supplement: Supplementary file 2 — Supplementary information for the Supplementary Video [file 41598_2019_42397_MOESM2_ESM.docx]

SUPPLEMENTARY INFORMATION for

**One-piece polarizing interferometer for ultrafast spectroscopic polarimetry**

Daesuk Kim^1,^**^*^** and Vamara Dembele^1^

*^1^Division of Mechanical System Engineering, Chonbuk National University, Jeonju 54896, Republic of Korea*

*****Corresponding author: [*dashi.kim@jbnu.ac.kr*](mailto:dashi.kim@jbnu.ac.kr)

Here, we provide a video to show the ultrafast dynamic spectro-polarization measurement capability of the proposed new concept based on a one-piece polarizing interferometer.

**Dynamic measurement capability of Δ*(k)***

The video attached as a supplement material (Visualization) shows that we can display a live spectral phase Δ(k) for the entire visible range dynamically by varying the QWP rotation angle as illustrated in Fig. 7 in the main text.


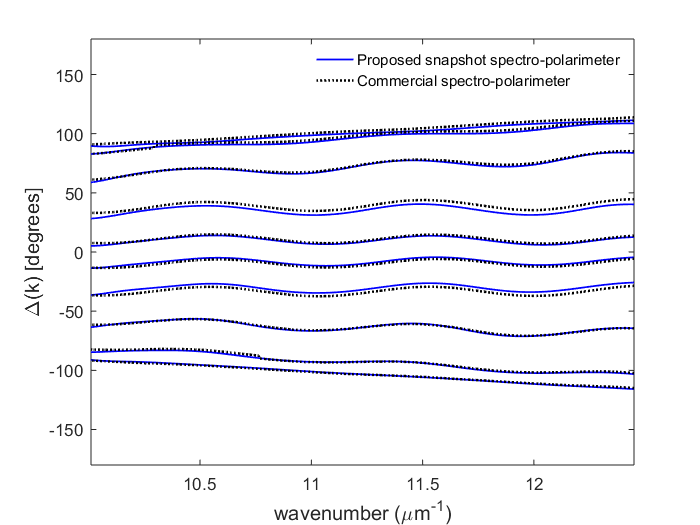


**Figure 1** Dynamic Δ*(k)* extraction while the optic axis angle of the QWP is varied from -45° to 45° (solid line: proposed snapshot spectro-polarimeter, and dotted line: commercial spectro-polarimeter).

Since a linear polarization state is generated at the output of the one-piece interferometer right before the QWP, the Δ(k) becomes around zero when the optic axis of the QWP aligns parallel to the polarization direction of the linear polarization generated. We observed the sinusoidally oscillating phenomenon resulting from the spectral characteristic of the QWP. The spectral phase Δ(k) gets close to around 90 degrees since right-circular polarization state is generated when the optic axis of the QWP is rotated by +45° from the first position as shown in the video. Likewise, the spectral phase Δ(k) approaches to -90 degrees for the left-circular polarization generation when the optic axis of the QWP is rotated by -45°. Note that we can measure the Δ(k) over 1,000 wavelengths in real time speed while maintaining high precision and accuracy.
